# A rare case of adult intestinal malrotation: A case report

**DOI:** 10.1016/j.ijscr.2025.110848

**Published:** 2025-01-06

**Authors:** Alazar Berhe Aregawi, Teketel Tadesse Geremew, Abel Tesfaye Legese, Teferi Tesfaye Bahru

**Affiliations:** a Department of Surgery, Hawassa University Comprehensive Specialized Hospital, Hawassa, Sidama, Ethiopia; b Department of Pathology, Hawassa University Comprehensive Specialized Hospital, Hawassa, Sidama, Ethiopia; c Department of surgery, Eka Kotebe General Hospital, Addis Ababa, Ethiopia; d Department of Infection Prevention and Patient Safety, Eka Kotebe General Hospital, Addis Ababa, Ethiopia

**Keywords:** Malrotation, Abdominal CT, Mid-gut volvulus, Adult intestinal obstruction, Ladd's procedure

## Abstract

**Introduction and importance:**

Intestinal malrotation is a congenital disorder resulting from the failure of the normal embryologic fetal sequence of bowel rotation and fixation. Adult midgut malrotation is extremely uncommon, with incidence estimates ranging from 0.0001 % to 0.19 %. In adults, there is a slight female predominance. Mid gut volvulus leads to obstruction and warrants surgical intervention. This case is significant because there are few case reports around the world.

**Case presentation:**

An 18-year-old Ethiopian man presented with cramping abdominal pain that worsened over a one-month period. He had been experiencing pain since early childhood, which worsened a year before his presentation. CT scan of the abdomen was consistent with malrotation and was treated surgically.

**Clinical discussion:**

Diagnosing intestinal malrotation in adults is difficult due to the variety and nonspecificity of the symptoms. Delays in diagnosis may lead to acute complications and poorer operative outcomes.

**Conclusion:**

This case report will raise awareness among physicians about the possibility of this rare condition affecting adults. As a result, when a patient presents with chronic gastrointestinal symptoms, they have a high index of suspicion and inform them that an abdominal CT should be performed in these cases, and surgery is the primary treatment option.

## Highlights

• Intestinal malrotation is a congenital disorder resulting from the failure of the normal embryologic fetal sequence of bowel rotation and fixation.
• Adult midgut malrotation is extremely uncommon, with incidence estimates ranging from 0.0001% to 0.19%.
• Mid gut malrotation leads to obstruction and warrants surgical intervention.

## 1 Introduction

Mid-gut malrotation is a congenital anomaly characterized by a defect in the normal embryonic developmental rotation of the gut, resulting in potential obstruction and presenting either acutely or with chronic intermittent gastrointestinal symptoms (1,2). During embryonic development, the digestive tract undergoes a complex process involving a 90-degree counterclockwise rotation followed by a 270-degree counterclockwise rotation within the abdominal cavity. More than 90 % of affected individuals will manifest symptoms before their first birthday (3). About 1 in 500 live babies are affected by the disease.

In 75–85 % of these patients, the diagnosis is made in infancy, and adult midgut malrotation is extremely rare, with incidence estimates ranging from 0.0001 % to 0.19 % (4). In adults, there is a slight female predominance. The diagnosis of adult malrotation poses significant challenges; patients frequently present with subtle obstructive symptoms, leading to potential misdiagnosis and necessitating a high index of suspicion. (1,3,4). Timely diagnosis and treatment are imperative to prevent severe complications such as small bowel necrosis. Several modalities are employed for diagnosis, including barium tests, computed tomography scans, angiography, and emergency laparotomy (1). The standard of treatment of malrotation, regardless of the age of the patient is the Ladd's procedure which was first described by Dr. Ladd in 1936 (1,2,5).

This report describes a case of adult midgut malrotation presenting with chronic obstructive symptoms in an 18-year-old Ethiopian man.

This case report is written following the SCARE criteria (6).

## 2 Case presentation

We present an 18-year-old Ethiopian man with crampy abdominal pain that worsened over a one-month period. He had been suffering from this pain since early childhood, and it worsened in the year leading up to his presentation. The pain was limited to the right upper quadrant and periumbilical area. He had a history of intermittent vomiting of ingested matter, which was sometimes mixed with bilious matter; he also had intermittent constipation and abdominal distension of similar duration. He had repeatedly visited health facilities but saw no improvement. A year ago, he was evaluated and offered an exploratory laparotomy at a nearby hospital, but he declined. He had no history of abdominal surgery. He had no previous or current use of long-term medications, alcohol, or tobacco.

## 3 Physical examination

### 3.1 General appearance

Acutely sick looking, Vital signs Pulse rate: 90 beats per minute Respiratory rate: 18 Breaths, Temperature: 37.1 °C, Blood pressure: 100/70 mmHg.

On head and neck examination: He had a Pink conjunctiva and non-icteric sclera;

On abdominal examination: abdomen moved symmetrically with respiration, there was visible peristalsis more on the right upper quadrant and epigastric area, bowel sounds were normal: there was no area of tenderness. The digital rectal examination was normal.

## 4 Investigations on imaging abdomino-pelvic CT with IV contrast

**Fig. 1:**
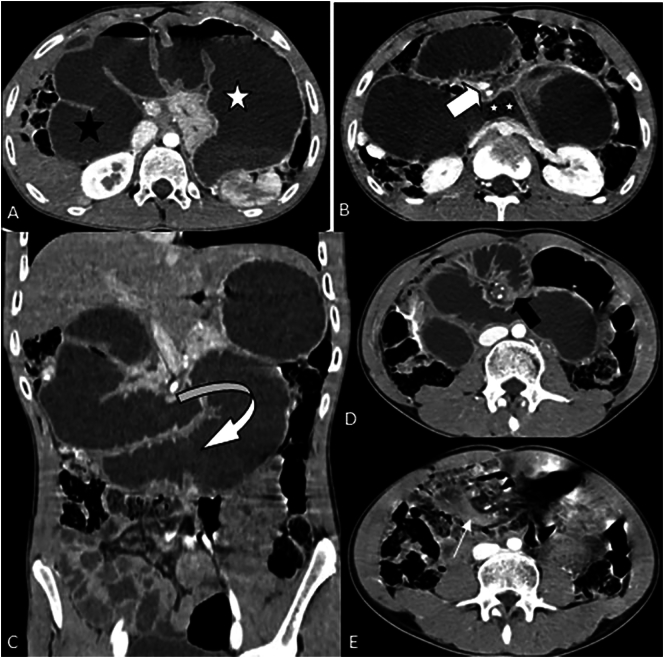
a-e: CT scan slices shows that there was a markedly dilated stomach, duodenum, and jejunum filled with fluid, the largest measuring 6.2 cm. The dilated small bowel abruptly transitions into the mid abdomen with a swirled appearance of mesenteric vessels (SMA and SMV). There was a distally collapsed small bowel to the point of the swirled sign in keeping with mid-gut volvulus. No abnormal wall thickening nor mass lesion.

Showed that there was a markedly dilated stomach, duodenum, and jejunum filled with fluid, the largest measuring 6.2 cm. The dilated small bowel abruptly transitions into the mid abdomen with a swirled appearance of mesenteric vessels (SMA and SMV). There was a distally collapsed small bowel to the point of the swirled appearance proximally. No abnormal wall thickening nor mass lesion were seen. The CT scan slice figure were shown below (Fig. 1: a,b,c,d,e). The conclusion of the abdominal CT was mid gut volvulus.

Based on the clinical and imaging evidence presented above, the patient was admitted with the possibility of having adult intestinal malrotation, and an exploratory laparotomy was scheduled for the following morning.

## 5 Intra operative finding

**Fig. 2 & 3:**
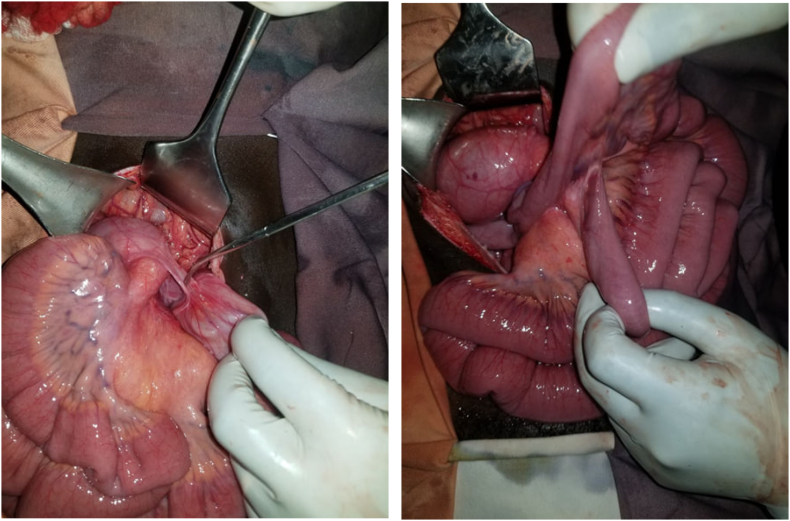
Intraoperative pictures of band adhesion and dilated proximal bowel.

Intra operatively the duodenum was hugely enlarged with a possible ladd's band extending to the transverse colon and obstructing dense adhesions at the duodeno-jejunal junction. T he rest of the small bowel was collapsed and localized to the right lower quadrant rotated 360° clock/counter clockwise direction (Fig. 2 & 3). Even if the abdominal CT suggested of a possible mid gut volvulus, we did not identify that.

**Fig. 4 & 5:**
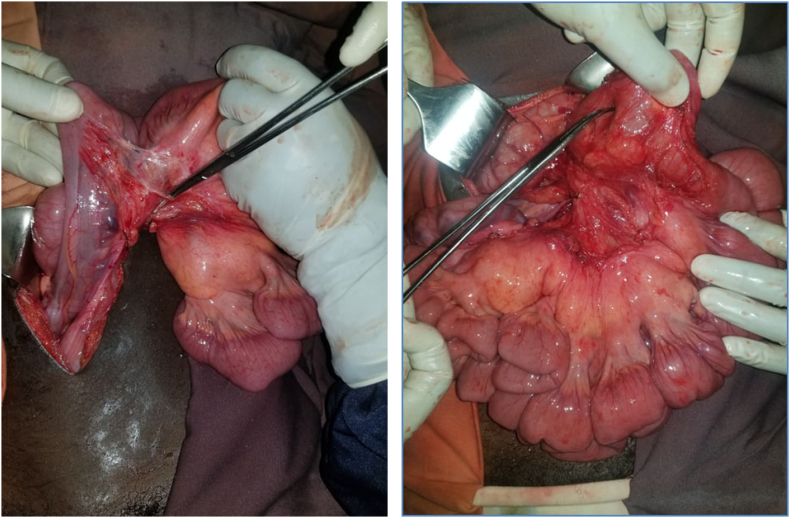
Intraoperative picture of small bowel mesentery widening and adenolysis.

The cecum & ascending colon resided along the right upper paracolic gutter with peritoneal attachment to the lateral abdominal wall (Fig. 2 & 3). The base of small bowel mesentery was significantly narrowed (Fig. 2–4).

Ladd's procedure with division of the band and release of the dense adhesions at the duodeno-jejunal junction was done; upon releasing, there was a significant jejunal peristalsis and jejunum filled with gastrointestinal content (Fig. 4 & 5). The small bowel mesentery was widened, appendectomy done and finally the small intestine was positioned at the right paracolic gutter (Fig. 4 & 5).

Finally, the wound closed in layers after the count was declared correct.

The patient had an uneventful post-operative course and had experienced no further symptoms when reviewed at 3 months.

## 6 Discussion

Small-bowel malrotation is a congenital defect in which the fetal intestines do not completely or at all rotate around the axis of the superior mesenteric artery during fetal development. (7). More specifically, the cecum is still linked to the right side of the abdominal wall by peritoneal fibrous bands known as Ladd's bands, the duodenum runs down the right side of the abdomen, and the Treitz ligament fails to cross the mid-line to the left side (8).

Intestinal malrotation is much more common in the pediatric population than in adults and is associated with higher morbidity and mortality rates. Hence, it is essential to take this into account when making a differential diagnosis for unexplained abdominal pain (9,10,13).

The symptoms of intestinal malrotation are varied and nonspecific, with no typical set of manifestations (1,2). The most common symptom is abdominal pain. The pain can be intermittent or persistently aching. Vomiting is also frequently present, but not necessarily bilious and abdominal pain and vomiting may be intermittent. Other less common presentations include failure to thrive, solid food intolerance, malabsorption, chronic diarrhea from protein-losing enteropathy, pancreatitis, peritonitis, biliary obstruction, motility disorders, and chylous ascites (1,12,13). In adults, the symptoms may mimic other gastrointestinal disorders, such as irritable bowel syndrome or peptic ulcer disease (14). Notably, older patients are more likely to present with atypical symptoms (15). Our patient presented with a long standing crampy abdominal pain, intermittent vomiting of ingested matter mixed with bilious matter, intermittent constipation, and abdominal distension.

Upper gastrointestinal (UGI) series, which is often used in pediatrics, continues to be the current criterion standard for diagnosis of intestinal malrotation. However, CT scans for adult patients, particularly when combined with intravenous and oral contrast, have a higher diagnostic value, and some studies advise using CT as the primary option for adults with suspected malrotation ([16], [17], [18]). Specific findings, such as abnormal positioning of the superior mesenteric vein and duodenojejunal junction, aid in diagnosis. Classic features of malrotation on abdominal CT include a right-sided small bowel (98.2 %), a left-sided cecum (12 %), an inverse relationship between SMA and the superior mensenteric vein (SMV) (only 8.4 %), and aplasia of the uncinate process. (1,14,19). The inverse positions of SMV in the setting of malrotation were first described by Nichols and Li. It refers to the superior mesenteric vein lying to the left of the SMA instead of to the right (20). Intestinal malrotation can shorten the mesenteric root and narrow the suspensory pedicle of the gut. The shortened mesentery allows the small bowel and mesentery to twist around the narrowed pedicle of SMA with midgut volvulus, which may be seen on CT as the “whirlpool sign.” (20). Our patient has CT scan with iv contrast shows dilated small bowel abruptly transitions into the mid abdomen with a swirled appearance of mesenteric vessels (SMA and SMV) which is consistent with malrotation with mid-gut volvulus, despite the fact that the volvulus was not identified during surgery.

Patients with chronic symptoms attributable to malrotation often require surgical correction for symptom resolution (1,3). Both laparotomy and laparoscopy are feasible techniques with low complication rates (2). Laparoscopic Ladd's procedure serves as an alternative to open surgery for treating chronic symptoms ([21], [22], [23]). However, controversy exists regarding surgical management in asymptomatic or minimally symptomatic patients (11,22). For our patient exploratory laparotomy and Ladd's procedure was done as the patient had chronic symptoms attributable to malrotation.

Delays in diagnosis may lead to acute complications and poorer surgical outcomes. (11,23,24). Postoperative complications are more common in adults, highlighting the importance of early recognition and management (11,[25], [26], [27]). The malrotated gut is prone to volvulus, leading to bowel obstruction and potential gangrene (28).

## 7 Conclusions

Intestinal malrotation in adults presents with diverse symptoms and poses diagnostic challenges. Malrotation presenting in adults is a rare presentation of the condition and diagnosis is difficult clinically. Early imaging-based diagnosis and prompt intervention are the cornerstones of improving patient outcomes. The Ladd procedure is a safe and effective surgical technique for treating intestinal malrotation.

## Abbreviations

UGI: Upper gastrointestinal
SMA: Superior mesenteric artery
SMV: Superior mesenteric Vein
CT: Computed tomography

## Author contribution

Alazar Berhe Aregawi, MD - Study concept and design, writing the paper, literature review and editing and critical review of the paper

Teketel Tadesse Geremew, MD - literature review of the paper, writing and drafting the paper, editing and critical review of the paper

Abel Tesfaye Legese and Teferi Tesfaye Bahru, MD - Involved in acquisition of data, literature review of the paper, writing and drafting the paper

## Patient consent

The report was done with patient's consent.

## Ethics approval

Written informed consent was obtained from the patient for publication and any accompanying images. A copy of the written consent is available for review by the Editor-in-Chief of this journal on request.

## Guarantor

Teketel Tadesse Geremew, MD

## Funding

No funding was provided for this case report.

## Declaration of competing interest

The authors declare that there are no conflicts of interest on this case report.
